# Supplementary material for: SARS-CoV-2 Droplet and Airborne Transmission Heterogeneity
Source: J Clin Med. 2022 May 6;11(9):2607. doi: 10.3390/jcm11092607 (PMC9099777; doi:10.3390/jcm11092607)
Supplement: Supplementary file 1 [file jcm-11-02607-s001.zip › jcm-1675874-supplementary.pdf]

SARS-CoV-2 droplet and airborne transmission heterogeneity

Marta Baselga[1], Antonio Güemes[1,2], Juan José Alba[1,3], Alberto J. Schuhmacher[1,4*]

[1] Institute for Health Research Aragon (IIS Aragón), 50009 Zaragoza, Spain

[2] Department of Surgery, University of Zaragoza, 50009 Zaragoza, Spain

[3] Department of Infectious Diseases, Hospital Clínico Universitario Lozano Blesa, 50009 Zaragoza, Spain

[4] Fundación Agencia Aragonesa para la Investigación y el Desarrollo (ARAID), 50018 Zaragoza, Spain

* Correspondence: ajimenez@iisaragon.es

Supplementary Materials

Table S1. Partial sequencing of the viral genome from aerosol-subgroup patients. These sequences were compared with the reference sequences of the main VOCs using Clustal Omega software (RRID: SCR_001591). All samples Variant of Concern Omicron BA.1 (OL672836). Patient 1´s sequence also present a silent mutation 21953 C>G

**Patient 1**	GTCTCTAGTCAGTGTGTTAATCTTACAACCAGAACTCAATTACCCCCTGCATACAC<br>TAATTCTTTCACACGTGGTGTTTATTACCCTGACAAAGTTTTCAGATCCTCAGTTT-<br>TACATTCACTCAGGACTTGTTCTTACCTTTCTTTTCCAATGTTACTTGGTTCCATGT-<br>TATCTCTGGGACCAATGGTACTAAGAGGTTTGATAACCCTGTCCTACCATT-<br>TAATGATGGTGTTTATTTTGCTTCCATTGAGAAGTCTAACATAATAAGAGGCTGGATTTTT-<br>GGTACTACTTTAGATTCGAAGACCCAGTCCCTACTTATTGTTAATAACGCTACTAATGTT-<br>GTTATTAAAGTCTGTGAATTTCAATTTTGTAATGATCCATTTTTGGACCACAAAAACAA-<br>CAAAAGTTGGATGGAAAGTGAGTTCAGAGTTTATTCTAGTGCGAATAATTGCACTTTT-<br>GAATATGTCTCTCAGCCTTTTCTTATGGACCTTGAAGGAAAACAGGGTAATTTCAA-<br>AAATCTTAGGGAATTTGTGTTTAAGAATATTGATGGTTATTTTAAAATATATTCTAAGCA-<br>CACGCCTATTATAGTGCGTGAGCCAGAAGATCTCCCTCAGGGTTTTTCGGCTTTAGAAC-<br>CATTGGTAGATTTGCCAATAGGTATTAACATCACTAGGTTTCAAACTTTACTTGCTTTACA-<br>TAGAAGTTATTTGACTCCTGGTGATTCTTCTTCAGG
**Patient 2**	CTCTAGTCAGTGTGTTAATCTTACAACCAGAACTCAATTACCCCCTGCATACAC-<br>TAATTCTTTCACACGTGGTGTTTATTACCCTGACAAAGTTTTCAGATCCTCAGTTT-<br>TACATTCAACTCAGGACTTGTTCTTACCTTTCTTTTCCAATGTTACTTGGTTCCATGT-<br>TATCTCTGGGACCAATGGTACTAAGAGGTTTGATAACCCTGTCCTACCATT-<br>TAATGATGGTGTTTATTTTGCTTCCATTGAGAAGTCTAACATAATAAGAGGCTGGATTTTT-<br>GGTACTACTTTAGATTCGAAGACCCAGTCCCTACTTATTGTTAATAACGCTACTAATGTT-<br>GTTATTAAAGTCTGTGAATTTCAATTTTGTAATGATCCATTTTTGGACCACAAAAACAA-<br>CAAAAGTTGGATGGAAAGTGAGTTTAGAGTTTATTCTAGTGCGAATAATTGCACTTTT-<br>GAATATGTCTCTCAGCCTTTTCTTATGGACCTTGAAGGAAAACAGGGTAATTTCAA-<br>AAATCTTAGGGAATTTGTGTTTAAGAATATTGATGGTTATTTTAAAATATATTCTAAGCA-<br>CACGCCTATTATAGTGCGTGAGCCAGAAGATCTCCCTCAGGGTTTTTCGGCTTTAGAAC-<br>CATTGGTAGATTTGCCAATAGGTATTAACATCACTAGGTTTCAAACTTTACTTGCTTTACA-<br>TAGAAGTTATTTGACTCCTGGTGATTCTTCTTCAG
**Patient 3**	TCTAGTCAGTGTGTTAATCTTACAACCAGAACTCAATTACCCCCTGCATACACTAA<br>TTCTTTCACACGTGGTGTTTATTACCCTGACAAAGTTTTCAGATCCTCAGTTTTACATT-<br>CAACTCAGGACTTGTTCTTACCTTTCTTTTCCAATGTTACTTGGTTCCATGTTATCTCTGG-<br>GACCAATGGTACTAAGAGGTTTGATAACCCTGTCCTACCATTTAATGATGGTGTTTATTTT-<br>GCTTCCATTGAGAAGTCTAACATAATAAGAGGCTGGATTTTTGGTACTACTTTA-<br>GATTCGAAGACCCAGTCCCTACTTATTGTTAATAACGCTACTAATGTTGTTAT-<br>TAAAGTCTGTGAATTTCAATTTTGTAATGATCCATTTTTGGACCACAAAAACAACAA-

	AAGTTGGATGGAAAGTGAGTTCAGAGTTTATTCTAGTGCGAATAATTGCACTTTTGAATA-TGTCTCTCAGCCTTTTCTTATGGACCTTGAAGGAAAACAGGGTAATTTCAAAAATCT-TAGGGAATTTGTGTTTAAGAATATTGATGGTTATTTTAAAATATATTCTAAGCACACGCC-TATTATAGTGCGTGAGCCAGAAGATCTCCCTCAGGGTTTTTCGGCTTTAGAACCATTGGTA-GATTTGCCAATAGGTATTAACATCACTAGGTTTCAAACTTTACTTGCTTTACATAGAAGT-TATTTGACTCCTGGTGATTCTTCTTCAG
**Patient 4**	ATCTTACAACCAGAACTCAATTACCCCCTGCATACACTAATTCTTTCACACGTGG TGTT-TATTACCCTGACAAAGTTTTCAGATCCTCAGTTTTACATTCAACTCAGGACTTGTTCTTA CCTTTCTTTTCCAATGTTACTTGGTTCCATGTTATCTCTGGGACCAATGGTACTAAGA-GGTTTGATAACCCTGTCCTACCATTTAATGATGGTGTTTATTTTGCTTCCATTGAGAA-GTCTAACATAATAAGAGGCTGGATTTTTGGTACTACTTTAGATTCGAAGACCCA-GTCCCTACTTATTGTTAATAACGCTACTAATGTTGTTATTAAAGTCTGTGAATTTCAA-TTTTGTAATGATCCATTTTTGGACCACAAAAACAACAAAAGTTGGATGGAAAGTGAGTT-CAGAGTTTATTCTAGTGCGAATAATTGCACTTTTGAATATGTCTCTCAGCCTTTTCTTATG-GACCTTGAAGGAAAACAGGGTAATTTCAAAAATCTTAGGGAATTTGTGTTTAAGAATATT-GATGGTTATTTTAAAATATATTCTAAGCACACGCCTATTATAGTGCGTGAGCCAGAA-GATCTCCCTCAGGGTTTTTCGGCTTTAGAACCATTGGTAGATTTGCCAATAGGTATTAACA-TCACTAGGTTTCAAACTTTACTTGCTTTACATAGAAGTTATTT-GACTCCTGGTGATTCTTCTTCAGG
**Patient 5**	TAGTCAGTGTGTTAATCTTACAACCAGAACTCAATTACCCCCTGCATACACTAATTCTTT-CACACGTGGTGTTTATTACCCTGACAAAGTTTTCAGATCCTCAGTTTTACATTCAA CTCA-GGACTTGTTCTTACCTTTCTTTTCCAATGTTACTTGGTTCCATGTTATCTCTGGGACCAA-TGGTACTAAGAGGTTTGATAACCCTGTCCTACCATTTAATGATGGTGTTTATTTTGCTTC-CATTGAGAAGTCTAACATAATAAGAGGCTGGATTTTTGGTACTACTTTAGATTCGAAGAC-CCAGTCCCTACTTATTGTTAATAACGCTACTAATGTTGTTATTAAAGTCTGTGAATTTCAA-TTTTGTAATGATCCATTTTTGGACCACAAAAACAACAAAAGTTGGATGGAAAGTGAGTT-CAGAGTTTATTCTAGTGCGAATAATTGCACTTTTGAATATGTCTCTCAGCCTTTTCTTATG-GACCTTGAAGGAAAACAGGGTAATTTCAAAAATCTTAGGGAATTTGTGTTTAAGAATATT-GATGGTTATTTTAAAATATATTCTAAGCACACGCCTATTATAGTGCGTGAGCCAGAA-GATCTCCCTCAGGGTTTTTCGGCTTTAGAACCATTGGTAGATTTGCCAATAGGTATTAACA-TCACTAGGTTTCAAACTTTACTTGCTTTACATAGAAGTTATTT-GACTCCTGGTGATTCTTCTTCAG
**Patient 20**	GTCTCTAGTCAGTGTGTTAATCTTACAACCAGAACTCAATTACCCCCTGCATAC AC-TAATTCTTTCACACGTGGTGTTTATTACCCTGACAAAGTTTTCAGATCCTCAGTTT-TACATTCAACTCAGGACTTGTTCTTACCTTTCTTTTCCAATGTTACTTGGTTCCATGT-TATCTCTGGGACCAATGGTACTAAGAGGTTTGATAACCCTGTCCTACCATT-TAATGATGGTGTTTATTTTGCTTCCATTGAGAAGTCTAACATAATAAGAGGCTGGATTTTT-GGTACTACTTTAGATTCGAAGACCCAGTCCCTACTTATTGTTAATAACGCTACTAATGTT-GTTATTAAAGTCTGTGAATTTCAATTTTGTAATGATCCATTTTTGGACCACAAAAACAA-CAAAAGTTGGATGGAAAGTGAGTTCAGAGTTTATTCTAGTGCGAATAATTGCACTTTT-GAATATGTCTCTCAGCCTTTTCTTATGGACCTTGAAGGAAAACAGGGTAATTTCAA-AAATCTTAGGGAATTTGTGTTTAAGAATATTGATGGTTATTTTAAAATATATTCTAAGCA-CACGCCTATTATAGTGCGTGAGCCAGAAGATCTCCCTCAGGGTTTTTCGGCTTTAGAAC-CATTGGTAGATTTGCCAATAGGTATTAACATCACTAGGTTTCAAACTTTACTTGCTTTACA-TAGAAGTTATTTGACTCCTGGTGATTCTTCTTC
**Patient 21**	CTAGTCAGTGTGTTAATCTTACAACCAGAACTCAATTACCCCCTGCATACACTAAT TCTTTCACACGTGGTGTTTATTACCCTGACAAAGTTTTCAGATCCTCAGTTTTACATTCAA-CTCAGGACTTGTTCTTACCTTTCTTTTCCAATGTTACTTGGTTCCATGTTATCTCTGGGAC-CAATGGTACTAAGAGGTTTGATAACCCTGTCCTACCATTTAATGATGGTGTTTATTTT-GCTTCCATTGAGAAGTCTAACATAATAAGAGGCTGGATTTTTGGTACTACTTTA-GATTCGAAGACCCAGTCCCTACTTATTGTTAATAACGCTACTAATGTTGTTAT-TAAAGTCTGTGAATTTCAATTTTGTAATGATCCATTTTTGGACCACAAAAACAACAA-AAGTTGGATGGAAAGTGAGTTCAGAGTTTATTCTAGTGCGAATAATTGCACTTTTGAATA-TGTCTCTCAGCCTTTTCTTATGGACCTTGAAGGAAAACAGGGTAATTTCAAAAATCT-TAGGGAATTTGTGTTTAAGAATATTGATGGTTATTTTAAAATATATTCTAAGCACACGCC-TATTATAGTGCGTGAGCCAGAAGATCTCCCTCAGGGTTTTTCGGCTTTAGAACCATTGGTA-

	GATTTGCCAATAGGTATTAACATCACTAGGTTTCAAACTTTACTTGCTTTACATAGAAGT-<br>TATTTGACTCCTGGTGATTCTTCTTCAGGTT
**Patient 22**	TCTAGTCAGTGTGTTAATCTTACAACCAGAACTCAATTACCCCCTGCATACACTAA<br>TTCTTTCACACGTGGTGTTTATTACCCTGACAAAGTTTTCAGATCCTCAGTTTTACATT-<br>CAACTCAGGACTTGTTCTTACCTTTCTTTTCCAATGTTACTTGGTTCCATGTTATCTCTGG-<br>GACCAATGGTACTAAGAGGTTTGATAACCCTGTCCTACCATTTAATGATGGTGTTTATTTT-<br>GCTTCCATTGAGAAGTCTAACATAATAAGAGGCTGGATTTTTGGTACTACTTTA-<br>GATTCGAAGACCCAGTCCCTACTTATTGTTAATAACGCTACTAATGTTGTTAT-<br>TAAAGTCTGTGAATTTCAATTTTGTAATGATCCATTTTTGGACCACAAAAACAACAA-<br>AAGTTGGATGGAAAGTGAGTTCAGAGTTTATTCTAGTGCGAATAATTGCACTTTTGAATA-<br>TGTCTCTCAGCCTTTTCTTATGGACCTTGAAGGAAAACAGGGTAATTTCAAAAATCT-<br>TAGGGAATTTGTGTTTAAGAATATTGATGGTTATTTTAAAATATATTCTAAGCACACGCC-<br>TATTATAGTGCGTGAGCCAGAAGATCTCCCTCAGGGTTTTTCGGCTTTAGAACCATTGGTA-<br>GATTTGCCAATAGGTATTAACATCACTAGGTTTCAAACTTTACTTGCTTTACATAGAAGT-<br>TATTTGACTCCTGGTGATTCTTCTTCAGG

Table S2. Viral load detected in aerosols per m^3 of air using different methods.

Method	*Viral load (copies/m^3) by RT-qPCR*	*Patients*	*Particle diameter*	*Ref.*
NIOSH BC 251 Ciclon<br>Time: 4 hours<br>Flow: 3.5 L/min<br>Volume: 5040 L<br>Medium: 15 mL (n/d)	1.84 E^3 - 3.38 E^3<br><br>Detection:<br>2/3 samples	2 patients (Ct 33.22; 18.45 and 20.11)	>4 μm<br>1–4 μm<br>(Not detected in <1 μm samples)	[1]
SASS 2300 Wetted Wall Cyclone<br>Time: 30 min<br>Flow: 300 L/min<br>Volume: 9000 L<br>Medium: n/d (n/d)	5.2 E^2 - 1.4 E^3<br><br>Detection:<br>UCI: 54/124 samples<br>GW: 9/114 samples	ICU 15 pat.<br>GW 24 pat.	n/d	[2]
Sartorius MD8<br>Time: 15 min<br>Flow: 50 L/min<br>Volume: 750 L<br>Medium: gelatin filter	2.42 E^3<br><br>Detection:<br>121/163 samples	3 patients and quarantin unit spaces	n/d	[3]
Coriolis μ<br>Time: 10 min<br>Flow: 100 L/min<br>Volume: 1000 L<br>Medium: 5 mL (DMEM)	5.05 E^2 – 5.15 E^2<br><br>Detection:<br>14/31 samples	Tracheostomy procedures, admissions ward, ICU and general area	n/d	[4]
VIVAS (condensation method)<br>Time: 1 hour<br>Flow: 6.5 L/min<br>Volume: 390 L<br>Medium: 1.5 mL (PBS)	8.7 E^3<br><br>Detection:<br>1/2 samples	Respiratory infection evaluation area	n/d	[5]
SKC biosampler<br>Time: 3 hours<br>Flow: 12 L/min<br>Volume: 2160 L<br>Medium: 15 mL (DMEM)	n/d<br><br>Detection:<br>2/14 samples, only in ICU areas	ICU areas (10 patients), hospital entrance and hall	0.25 and 32 μm particle size detection with aerosol spectrometer	[6]
Cascade impactor<br>Time: 48 hours<br>Flow: 5 L/min<br>Volume: 14400 L	5 - 5.1 E^1<br><br>Detection:<br>9/90 samples	COVID areas, close to ICU, nursing and workers workstations	≤ 2.5 μm,<br>2.5-10.0 μm and<br>> 10 μm	[7]
NIOSH W-15 cyclone<br>Time: 30 min<br>Flow: 3.5 L/min<br>Volume: 210 L	n/d	ICU and isolation ward	n/d	[8]

Medium: 15 mL (>4.0 μm), 1.5 mL (1.0-4.0 μm) and PTFE filter (<1.0 μm)<br><br>DingBlue sampler<br>Time: 30 min<br>Flow: 14 L/min<br>Volume: 420 L<br>Medium: 3 mL (VTM)	Detection:<br>UCI: 1/218 samples<br>IW: 9/182			
Coriolis μ<br>Time: 10 min<br>Flow: 300 L/min<br>Volume: 3000 L<br>Medium: 15 mL (PBS)<br>Sartorius MD8<br>Time: 10 min<br>Flow: 50 L/min<br>Volume: 500 L<br>Medium: gelatin filter	<10 – 4.6 E^2<br><br>Detection:<br>4/55 samples	ICU and non-ICU COVID settings	n/d	[9]
Coriolis μ<br>Time: 10 min<br>Flow: 100 L/min<br>Volume: 1000 L<br>Medium: 4 mL (PBS)	6.36 E^3 – 6.66 E^3<br><br>Detection:<br>2/44 samples	4 COVID areas and 7 non-COVID areas from an Hospital	n/d	[10]
Coriolis μ<br>Time: 10 min<br>Flow: 100 L/min<br>Volume: 1000 L<br>Medium: 5 mL (DMEM)	1.31 E^2 – 7.05 E^3<br><br>Detection:<br>2/31 samples	Emergency Department and hospital public areas	n/d	[11]
Coriolis μ<br>Time: 10 min<br>Flow: 150 L/min<br>Volume: 1500 L<br>Medium: 5 mL (VTM)	0.64 E^2 – 11.94 E^3<br><br>Detection:<br>ICU: 4/23 samples<br>WR: 7/92 samples<br>LTCR: 3/15<br>FR: 1/8	COVID rooms (WR), ICU areas and rooms in long-term care homes (LTCT) and facility rooms (FR)	n/d	[12]
Coriolis μ<br>Time: 10 min<br>Flow: 300 L/min<br>Volume: 3000 L<br>Medium: 15 mL (PBS)	n/d<br><br>Detection:<br>4/90 samples	COVID rooms	n/d	[13]
SKC biosampler impigner<br>Time: 30 min<br>Flow: 12.5 L/min<br>Volume: 375 L<br>Medium: 10 mL (PBS)	2.6 E^2 – 7.1 E^2<br><br>Detection:<br>16/86 samples	COVID rooms and other hospital areas	n/d	[14]
PM2.5 Filters<br>Time: 10 hours<br>Flow: 10 L/min<br>Volume: 6000 L	9.89 E^2 – 9.50 E^3<br><br>Detection:<br>3/12 samples	Public transport	n/d	[15]
Sartorius MD8<br>Time: 4, 6 and 18 hours<br>Flow: 10 L/min<br>Volume: 240, 260 and 1080 L<br>Medium: gelatin filter	<2.5 E^1 – 5.1 E^2<br><br>Detection:<br>11/100 samples	COVID patient rooms	n/d	[16]
WA-400 DingBlue Centrifugal<br>Time: 30 min<br>Flow: 400 L/min<br>Volume: 12000 L<br>Medium: 2 mL (PBS)	1.11 E^3 – 1.12 E^4<br><br>Detection:<br>ICU: 8/38 samples<br>TAC: 1/6 samples	ICU places and TAC rooms	n/d	[17]
Derenda PNS 16T-3.1<br>Time: 1.5 hours<br>Flow: 16.7 L/min	5.5 E^1 – 3.15 E^3<br><br>Detection:	AIIR	n/d	[18]

Volume: 1500 L<br>Medium: membrane filter	25/1544 samples			

Where, ICU = Intensive Care Unit; GW = General Ward; AIIR = Airborne Infectious Isolation Rooms.

Table S3. Some superspreading events. Modified from [19].

Setting	Number of sites	Total infected	Reference
Religious	2	4531	[20]
	3	150	[21]
	1	102	[22]
	2	33	[23]
	1	19	[24]
Worker dormitories	17	1690	[23]
Food processing plant	1	1029	[25]
	3	779	[26]
	1	534	[27]
	4	469	[28]
Work	2	34	[23]
	3	25	[29]
School	1	133	[30]
	1	130	[31]
	1	96	[32]
	1	26	[33]
Shopping	3	122	[23]
	3	44	[34]
Hospital	1	118	[20]
	1	54	[35]
	2	20	[36]
Bar	6	100	[36]
	1	6	[37]
	1	77	[38]
	1	16	[39]
	1	15	[40]
	4	106	[24]
Building Site	3	90	[23]
Conference	1	89	[41]
Meal	2	55	[23]
	2	19	[42]
Aircraft	1	26	[43]
Skiing	1	11	[44]
Bus	1	30	[45]

Table S4. Dose-response characterization using animal models. Modified from Karimzadeh et al.[46]

Host	*Dose (copies)*	*Infection rate*	*Inoculation method*	*Ref*
Ferret	221,359	6/6	Intranasal	[47]
Ferret	500	1/6	Intranasal	[48]
	50,000	6/6		
	500,000	6/6		
Ferret	420,000	4/4	Intranasal	[49]
Mice (hACE2)	70,000	7/19	Intranasal	[50]
Mice (hACE2)	400,000	3/3	Intranasal	[51]
	4,000,000	1/3	Intragastric	
Mice (hACE2)	630	2/2	Aerosols	[52]
Mice (hACE2)	21,000	24/24	Intranasal	[53]
Mice (hACE2)	100,000	11	Intranasal	[54]
Cynomolgus macaque	700,000	4/4	Intranasal, intratraqueal	[55]

Rhesus macaque	700,000	2/2	Intraocular	[56]
		1/1	Intrathecal	
		0/2	Intragastric	
Rhesus macaque	1,820,000	8/8	Intranasal, intrathecal, intraocular, oral	[57]
Rhesus macaque	11,000	3/3	Intranasal, intrathecal	[58]
	110,000	3/3	Intranasal, intrathecal	
	1,100,000	3/3	Intranasal, intrathecal	
Cynomolgus macaque	48,600	4/4	Aerosols	[59]
Rhesus macaque	28,700	4/4		
African Green Monkeys	38,000	3/3		
African Green Monkeys	500,000	6/6	Intranasal, intrathecal	[60]
African Green Monkeys	3,000,000	6/6	Intranasal	[61]
African Green Monkeys	2,000	2/2	Aerosols	[62]
	3,610,000	2/2	Intranasal, intrathecal intraocular, oral	
Syrian hamster	398,107	4/4	Intranasal, intraocular	[63]
	1,000		Intranasal, intraocular	
Syrian hamster	56,000	3/3	Intranasal	[64]
Syrian hamster	100,000	24/36	Intranasal	[65]
Syrian hamster	70,000	7/9	Intranasal	[66]

References

1. Chia, P.Y.; Coleman, K.K.; Tan, Y.K.; Ong, S.W.X.; Gum, M.; Lau, S.K.; Lim, X.F.; Lim, A.S.; Sutjipto, S.; Lee, P.H.; et al. Detection of Air and Surface Contamination by SARS-CoV-2 in Hospital Rooms of Infected Patients. *Nat. Commun.* **2020**, *11*, doi:10.1038/s41467-020-16670-2.

2. CHEN, G.M.; JI, J.J.; JIANG, S.; XIAO, Y.Q.; ZHANG, R.L.; HUANG, D.N.; LIU, H.; YU, S.Y. Detecting Environmental Contamination of Acute Respiratory Syndrome Coronavirus 2 (SARS-CoV-2) in Isolation Wards and Fever Clinics*. *Biomed. Environ. Sci.* **2020**, *33*, 943–947, doi:10.3967/bes2020.130.

3. Santarpia, J.L.; Rivera, D.N.; Herrera, V.L.; Morwitzer, M.J.; Creager, H.M.; Santarpia, G.W.; Crown, K.K.; Brett-Major, D.M.; Schnaubelt, E.R.; Broadhurst, M.J.; et al. Aerosol and Surface Contamination of SARS-CoV-2 Observed in Quarantine and Isolation Care. *Sci. Rep.* **2020**, *10*, 1–8, doi:10.1038/s41598-020-69286-3.

4. Zhou, A.J.; Otter, J.A.; Price, J.R.; Cimpeanu, C.; Garcia, M.; Kinross, J.; Boshier, P.R.; Mason, S.; Bolt, F.; Alison, H.; et al. Investigating SARS-CoV-2 Surface and Air Contamination in an Acute Healthcare 2 Setting during the Peak of the COVID-19 Pandemic in London. *medRxiv Prepr.* **2020**, 1–24.

5. Lednicky, J.; Shankar, S.; Elbadry, M.; Gibson, J.; Alam, M.; Stephenson, C.; EIGUREN, A.; Glenn, J.; Mavian, C.; Salemi, M.; et al. Collection of SARS-CoV-2 Virus from the Air of a Clinic Within a University Student Health Care Center and Analyses of the Viral Genomic Sequence. *Aerosol Air Qual. Res.* **2020**, *20*, 1167–1171, doi:10.4209/aaqr.2020.02.0202.Collection.

6. Kenarkoohi, A.; Noorimotlagh, Z.; Falahi, S.; Amarloei, A.; Abbas, S. Hospital Indoor Air Quality Monitoring for the Detection of SARS-CoV-2 (COVID-19) Virus. *Sci. Total Envionment* **2020**, *748*, 141324.

7. Stern, R.A.; Koutrakis, P.; Martins, M.A.G.; Lemos, B.; Dowd, S.E.; Sunderland, E.M.; Garshick, E. Characterization of Hospital Airborne SARS-CoV-2. *Respir. Res.* **2021**, *22*, 1–8, doi:10.1186/s12931-021-01637-8.

8. Lei, H.; Ye, F.; Liu, X.; Huang, Z.; Ling, S.; Jiang, Z.; Cheng, J.; Huang, X.; Wu, Q.; Wu, S.; et al. *SARS-CoV-2 Environmental Contamination Associated with Persistently Infected COVID-19 Patients*; 2020; Vol.

14; ISBN 0000000173148.

9. Moore, G.; Rickard, H.; Stevenson, D.; Aranega-Bou, P.; Pitman, J.; Crook, A.; Davies, K.; Spencer, A.; Burton, C.; Easterbrook, L.; et al. Detection of SARS-CoV-2 within the Healthcare Environment: A Multi-Centre Study Conducted during the First Wave of the COVID-19 Outbreak in England. *J. Hosp. Infect.* **2021**, *108*, 189–196, doi:10.1016/j.jhin.2020.11.024.

10. Gomes, P.; Gonçalves, J.; Isabel, A.; Lopes, B.; Esteves, N.A.; Emanuel, G.; Bamba, E.; Maria, S.; Branco, P.T.B.S.; Soares, R.R.G.; et al. Evidence of Air and Surface Contamination with SARS-CoV-2 in a Major Hospital in Portugal. **2022**.

11. Department of Infectious Disease, Imperial College London, London, UK, W. 1PG EVALUATION OF SARS-C o V-2 AIR CONTAMINATION IN HOSPITALS WITH CORIOLIS μ AIR SAMPLER / CONTEXT / PROTOCOL / RESULTS / CUSTOMER / CONCLUSION. **2020**, 2.

12. Mallach, G.; Kasloff, S.B.; Kovesi, T.; Kumar, A.; Kulka, R.; Krishnan, J.; Robert, B.; McGuinty, M.; den Otter-Moore, S.; Yazji, B.; et al. Aerosol SARS-CoV-2 in Hospitals and Long-Term Care Homes during the COVID-19 Pandemic. *PLoS One* **2021**, *16*, 1–17, doi:10.1371/journal.pone.0258151.

13. Winslow, R.L.; Zhou, J.; Windle, E.F.; Nur, I.; Lall, R.; Ji, C.; Millar, J.E.; Dark, P.; Naisbitt, J.; Simonds, A.; et al. SARS-CoV-2 Environmental Contamination from Hospitalised COVID-19 Patients Receiving Aerosol Generating Procedures. *Thorax* **2021**, thoraxjnl-2021-218035, doi:10.1101/2021.07.04.21259945.

14. Styczynski, A.; Hemlock, C.; Hoque, K.I.; Verma, R.; LeBoa, C.; Bhuiyan, M.O.F.; Nag, A.; Harun, M.G.D.; Amin, M.B.; Andrews, J.R. Ventilation and Detection of Airborne SARS-CoV-2: Elucidating High-Risk Spaces in Naturally Ventilated Healthcare Settings. *medRxiv* **2021**, 2021.06.30.21258984.

15. Moreno, T.; Pintó, R.M.; Bosch, A.; Moreno, N.; Alastuey, A.; Minguillón, M.C.; Anfruns-Estrada, E.; Guix, S.; Fuentes, C.; Buonanno, G.; et al. Tracing Surface and Airborne SARS-CoV-2 RNA inside Public Buses and Subway Trains. *Environ. Int.* **2021**, *147*, 106326, doi:10.1016/j.envint.2020.106326.

16. Dumont-Leblond, N.; Veillette, M.; Mubareka, S.; Yip, L.; Longtin, Y.; Jouvet, P.; Paquet Bolduc, B.; Godbout, S.; Kobinger, G.; McGeer, A.; et al. Low Incidence of Airborne SARS-CoV-2 in Acute Care Hospital Rooms with Optimized Ventilation. *Emerg. Microbes Infect.* **2020**, *9*, 2597–2605, doi:10.1080/22221751.2020.1850184.

17. Hu, J.; Lei, C.; Chen, Z.; Liu, W.; Hu, X.; Pei, R.; Su, Z.; Deng, F.; Huang, Y.; Sun, X.; et al. Distribution of Airborne SARS-CoV-2 and Possible Aerosol Transmission in Wuhan Hospitals, China. *Natl. Sci. Rev.* **2020**, *7*, 1865–1867.

18. Song, Z.G.; Chen, Y.M.; Wu, F.; Xu, L.; Wang, B.F.; Shi, L.; Chen, X.; Dai, F.H.; She, J.L.; Chen, J.M.; et al. Identifying the Risk of SARS-CoV-2 Infection and Environmental Monitoring in Airborne Infectious Isolation Rooms (AIIRs). *Virol. Sin.* **2020**, *35*, 785–792, doi:10.1007/s12250-020-00301-7.

19. Majra, D.; Benson, J.; Pitts, J.; Stebbing, J. SARS-CoV-2 (COVID-19) Superspreader Events. *J. Infect.* **2021**, *82*, 36–40.

20. Shim, E.; Tariq, A.; Choi, W.; Lee, Y.; Chowell, G. Transmission Potential and Severity of COVID-19 in South Korea. *Int. J. Infect. Dis.* **2020**, *93*, 339–344, doi:10.1016/j.ijid.2020.03.031.

21. Zauzmer, J.; Washington-Post 'Take It Very Seriously': Pastor at Arkansas Church Where 34 People Came down with Coronavirus Sends a Warning.

22. McKie, R.; TheObserver Did Singing Together Spread Coronavirus to Four Choirs?

23. Dashboard of the COVID-19 Virus Outbreak in Singapore.

24. Adam, D.C.; Wu, P.; Wong, J.Y.; Lau, E.H.Y.; Tsang, T.K.; Cauchemez, S.; Leung, G.M.; Cowling, B.J. Clustering and Superspreading Potential of SARS-CoV-2 Infections in Hong Kong. *Nat. Med.* **2020**, *26*, 1714–1719, doi:10.1038/s41591-020-1092-0.

25. DW Coronavirus: German Slaughterhouse Outbreak Crosses.

26. Cannon, A. Eu.Desmoinesregister.Com.

27. Adebayo, B. A Worker Infected 533 Others With Coronavirus at a Factory in Ghana, President Says.

28. Halliday, J.; TheGuardian Over 450 Covid-19 Cases Reported at Food Factories in England and Wales.

29. Park, S.Y.; Kim, Y.M.; Yi, S.; Lee, S.; Na, B.J.; Kim, C.B.; Kim, J. Il; Kim, H.S.; Kim, Y.B.; Park, Y.; et al. Coronavirus Disease Outbreak in Call Center, South Korea. *Emerg. Infect. Dis.* **2020**, *26*, 1666–1670, doi:10.3201/eid2608.201274.

30. Fontanet, A.; Tondeur, L.; Madec, Y.; Grant, R.; Besombes, C.; Jolly, N.; Pellerin, S.F.; Ungeheuer, M.-N.; Cailleau, I.; Kuhmel, L.; et al. Cluster of COVID-19 in Northern France: A Retrospective Closed Cohort Study. *SSRN Electron. J.* **2020**, 1–22, doi:10.2139/ssrn.3582749.

31. S., K.-O.; N., H.; I., E. Schools in Jerusalem Shut as Dozens of Students, Staff Test Positive for the Coronavirus.

32. Ministry of Health New Zeland, N. COVID-19 - Significant Clusters.

33. MOH News Highlights.

34. Cai, J.; Sun, W.; Huang, J.; Gamber, M.; Wu, J.; He, G. Indirect Virus Transmission in Cluster of COVID-19 Cases, Wenzhou, China, 2020. *Emerg. Infect. Dis.* **2020**, *26*, 1343–1345.

35. Chu, J.; Yang, N.; Wei, Y.; Yue, H.; Zhang, F.; Zhao, J.; He, L.; Sheng, G.; Chen, P.; Li, G.; et al. Clinical Characteristics of 54 Medical Staff with COVID-19: A Retrospective Study in a Single Center in Wuhan, China. *J. Med. Virol.* **2020**, *92*, 807–813, doi:10.1002/jmv.25793.

36. Sim, W. The Straits Times; Published 2020. Japan Identifies 15 Clusters as Covid-19 Cases.

37. LeTemps «Superspreader»: 300 Visiteurs d'un Club Zurichois En Quarantaine.

38. Stuff Coronavirus: Matamata Bar Owner on NZ's Biggest Covid-19 Cluster Outbreak.

39. Aljazeera After One Infected 16 at Berlin Nightclub, Coronavirus Fears Grow.

40. CNN How an Austrian Ski Resort Helped Coronavirus Spread Across Europe.

41. Marcelo, P.; O'brien, M. Cluster of Coronavirus Cases Tied to U.S. Biotech Meeting.

42. Sonja A. Rasmussen, MD, MS, J.C.S.; John C. Smulian Sonja A. Rasmussen MD, M.S.; Zhu, X.; Ge, Y.; Wu, T.; Zhao, K.; Chen, Y.; Wu, B.; Zhu, F.; Zhu, B.; et al. Secondary Attack Rate and Superspreading Events for SARS-CoV-2. *Lancet* **2020**, *365*, e47, doi:10.1056/NEJMoa2001316.Riou.

43. Orban, A. 26 Passengers Tested Positive for Covid-19 on an Emirates Flight to Hong Kong.

44. Zeichner, L.O.; Shoham, S.; Vazquez, J.; Reboli, A.; Betts, R. Cluster of COVID-19 in French Alps, 2020. **2014**, *2019*, 1–26.

45. Shen, Y.; Li, C.; Dong, H.; Wang, Z.; Martinez, L.; Sun, Z.; Handel, A.; Chen, Z.; Chen, E.; Ebell, M.H.; et al. Community Outbreak Investigation of SARS-CoV-2 Transmission among Bus Riders in Eastern China. *JAMA Intern. Med.* **2020**, *180*, 1665–1671, doi:10.1001/jamainternmed.2020.5225.

46. Karimzadeh, S.; Bhopal, R.; Huy, N.T. Review of Infective Dose, Routes of Transmission, and Outcome of COVID-19 Caused by the SARS-COV-2: Comparison with Other Respiratory Viruses. *Epidemiol. Infect.* **2021**, doi:10.1017/S0950268821000790.

47. Kim, Y. Il; Kim, S.G.; Kim, S.M.; Kim, E.H.; Park, S.J.; Yu, K.M.; Chang, J.H.; Kim, E.J.; Lee, S.; Casel, M.A.B.; et al. Infection and Rapid Transmission of SARS-CoV-2 in Ferrets. *Cell Host Microbe* **2020**, *27*, 704-709.e2, doi:10.1016/j.chom.2020.03.023.

48. Ryan, K.A.; Bewley, K.R.; Fotheringham, S.A.; Slack, G.S.; Brown, P.; Hall, Y.; Wand, N.I.; Marriott, A.C.; Cavell, B.E.; Tree, J.A.; et al. Dose-Dependent Response to Infection with SARS-CoV-2 in the Ferret Model and Evidence of Protective Immunity. *Nat. Commun.* **2021**, *12*, 1–13, doi:10.1038/s41467-

020-20439-y.

49. Richard, M.; Kok, A.; Meulder, D. De; Bestebroer, T.M.; Lamers, M.M.; Okba, N.M.A.; Vlissingen, M.F. Van; Rockx, B.; Haagmans, B.L.; Marion, P.G.; et al. SARS-CoV-2 Is Transmitted via Contact and via the Air between Ferrets. *Nat. Commun.* **2020**.

50. Bao, L.; Deng, W.; Huang, B.; Gao, H.; Liu, J.; Ren, L.; Wei, Q.; Yu, P.; Xu, Y.; Qi, F.; et al. The Pathogenicity of SARS-CoV-2 in HACE2 Transgenic Mice. *Nature* **2020**, *583*, 830–833, doi:10.1038/s41586-020-2312-y.

51. Sun, S.H.; Chen, Q.; Gu, H.J.; Yang, G.; Wang, Y.X.; Huang, X.Y.; Liu, S.S.; Zhang, N.N.; Li, X.F.; Xiong, R.; et al. A Mouse Model of SARS-CoV-2 Infection and Pathogenesis. *Cell Host Microbe* **2020**, *28*, 124-133.e4, doi:10.1016/j.chom.2020.05.020.

52. Bao, L.; Gao, H.; Deng, W.; Lv, Q.; Yu, H.; Liu, M.; Yu, P.; Liu, J.; Qu, Y.; Gong, S.; et al. Transmission of Severe Acute Respiratory Syndrome Coronavirus 2 via Close Contact and Respiratory Droplets among Human Angiotensin-Converting Enzyme 2 Mice. *J. Infect. Dis.* **2020**, *222*, 551–555, doi:10.1093/infdis/jiaa281.

53. Jiang, R. Di; Liu, M.Q.; Chen, Y.; Shan, C.; Zhou, Y.W.; Shen, X.R.; Li, Q.; Zhang, L.; Zhu, Y.; Si, H.R.; et al. Pathogenesis of SARS-CoV-2 in Transgenic Mice Expressing Human Angiotensin-Converting Enzyme 2. *Cell* **2020**, *182*, 50-58.e8, doi:10.1016/j.cell.2020.05.027.

54. Dinnon, H.; Leist, S.; Schäfer, A.; Edwards, C.; Martinez, D.; Montgomery, S.; West, A.; Yount, B.; Hou, Y.; Adams, L.; et al. A Mouse-Adapted SARS-CoV-2 Model for the Evaluation of COVID-19 Medical Countermeasures. *bioRxiv Prepr.* **2020**.

55. Rockx, B.; Kuiken, T.; Herfst, S.; Bestebroer, T.; Lamers, M.M.; Munnink, B.B.O.; De Meulder, D.; Van Amerongen, G.; Van Den Brand, J.; Okba, N.M.A.; et al. Comparative Pathogenesis of COVID-19, MERS, and SARS in a Nonhuman Primate Model. *Science (80-.).* **2020**, *368*, 1012–1015, doi:10.1126/science.abb7314.

56. Deng, W.; Bao, L.; Gao, H.; Xiang, Z.; Qu, Y.; Song, Z.; Gong, S.; Liu, J.; Liu, J.; Yu, P.; et al. Ocular Conjunctival Inoculation of SARS-CoV-2 Can Cause Mild COVID-19 in Rhesus Macaques. *Nat. Commun.* **2020**, *11*, 1–7, doi:10.1038/s41467-020-18149-6.

57. Munster, V.J.; Feldmann, F.; Williamson, B.N.; Doremalen, N. van; Pérez-Pérez, L.; Schulz, J.; Meade-White, K.; Okumura, A.; Callison, J.; Brumbaugh, B.; et al. Respiratory Disease and Virus Shedding in Rhesus Macaques Inoculated with SARS-CoV-2 1. *bioRxiv* **2020**.

58. Chandrashekar, A.; Liu, J.; Martino, A.J.; McMahan, K.; Mercad, N.B.; Peter, L.; Tostanosk, L.H.; Yu, J.; Maliga, Z.; Nekorchuk, M.; et al. SARS-CoV-2 Infection Protects against Rechallenge in Rhesus Macaques. *Science (80-.).* **2020**, *369*, 812–817, doi:10.1126/science.abc4776.

59. Johnston, S.C.; Ricks, K.M.; Jay, A.; Raymond, J.L.; Rossi, F.; Zeng, X.; Scruggs, J.; Dyer, D.; Frick, O.; Koehler, J.W.; et al. Development of a Coronavirus Disease 2019 Nonhuman Primate Model Using Airborne Exposure. *PLoS One* **2021**, *16*, 1–22, doi:10.1371/journal.pone.0246366.

60. Woolsey, C.; Borisevich, V.; Prasad, A.N.; Agans, K.N.; Deer, D.J.; Dobias, N.S.; Heymann, J.C.; Foster, S.L.; Levine, C.B.; Medina, L.; et al. Establishment of an African Green Monkey Model for COVID-19 and Protection against Re-Infection. *Nat. Immunol.* **2021**, *22*, 86–98, doi:10.1038/s41590-020-00835-8.

61. Cross, R.W.; Agans, K.N.; Prasad, A.N.; Borisevich, V.; Woolsey, C.; Deer, D.J.; Dobias, N.S.; Geisbert, J.B.; Fenton, K.A.; Geisbert, T.W. Intranasal Exposure of African Green Monkeys to SARS-CoV-2 Results in Acute Phase Pneumonia with Shedding and Lung Injury Still Present in the Early Convalescence Phase. *Virol. J.* **2020**, *17*, 1–12, doi:10.1186/s12985-020-01396-w.

62. Blair, R.; Vaccari, M.; Doyle, L.; Roy, C.; Russell, K.; Fahlberg, M.; Monjure, C.; Beddingfield, B.; Plante, K.; Plante, K.; et al. ARDS and Cytokine Storm in SARS-CoV-2 Infected Caribbean Vervets. *bioRxiv* **2021**.

63. Imai, M.; Iwatsuki-Horimoto, K.; Hatta, M.; Loeber, S.; Halfmann, P.J.; Nakajima, N.; Watanabe, T.; Ujie, M.; Takahashi, K.; Ito, M.; et al. Syrian Hamsters as a Small Animal Model for SARS-CoV-2 Infection and Countermeasure Development. *Proc. Natl. Acad. Sci. U. S. A.* **2020**, *117*, 16587–16595, doi:10.1073/pnas.2009799117.

64. Sia, S.F.; Yan, L.M.; Chin, A.W.H.; Fung, K.; Choy, K.T.; Wong, A.Y.L.; Kaewpreedee, P.; Perera, R.A.P.M.; Poon, L.L.M.; Nicholls, J.M.; et al. Pathogenesis and Transmission of SARS-CoV-2 in Golden Hamsters. *Nature* **2020**, *583*, 834–838, doi:10.1038/s41586-020-2342-5.

65. Osterrieder, N.; Bertzbach, L.D.; Dietert, K.; Abdelgawad, A. Age-Dependent Progression of SARS-CoV-2 Infection. *Viruses* **2020**, *12*, 1–11.

66. Schlottau, K.; Rissmann, M.; Graaf, A.; Schön, J.; Sehl, J.; Wylezich, C.; Höper, D.; Mettenleiter, T.C.; Balkema-Buschmann, A.; Harder, T.; et al. SARS-CoV-2 in Fruit Bats, Ferrets, Pigs, and Chickens: An Experimental Transmission Study. *The Lancet Microbe* **2020**, *1*, e218–e225, doi:10.1016/S2666-5247(20)30089-6.
